# Pavy on Food and Dietetics

**Published:** 1874-09

**Authors:** 


A TREATISE ON FOOD AND DIETETICS, *Physiologically and Therapeutically Considered.* By F. W. Pavy, M.D., F.R.S., Fellow of the Royal College of Physicians; Physician to, and Lecturer on, Physiology, at Guy's Hospital. Philadelphia: Henry C. Lea, 1874. Octavo, cloth, pp. 574. Price $4.75.

The great change, the entire revolution, rather, we ought to say, which has taken place in medical therapeutics in our day, demands a careful study of the whole subject of alimentation, and invests it with an importance and remedial value in the cure of disease which was but little recognized a third of a century ago. Then disease was an imprisoned monster, to be "jugulated" with the lancet, or expelled *vi et armis* from the human system by our drugs and medicaments. Now disease is an expression of something that is lacking, rather than something in excess, and the human body is to be nourished and sustained until the want can be supplied, or the problem of restoration be duly solved by the innate vital forces. Then, medical homilies were written in blood; now, in milk and oil. Then, *jugulare febrim* was the watchword; now, we have the "restorative system of medicine" of Reynolds, and the "conservative medicine" of Flint. Then, the lancet, the vial of calomel, and the ball of opium in the vest pocket sufficed; now, the butcher, the cook-stove and the flask of brandy are the reliance.

The work before us is entirely new, and, as a monogram, fills a hiatus in the professional library. In treats the whole subject of nutritive materials and their application to the nutrition of the body, including the chemistry and physiology of digestion and the assimilative processes. Its advent at this time will be hailed as auspicious, and we trust it will be widely read by our subscribers.

